# Editorial: Mechanisms of Neuronal Recovery in the Central Nervous System

**DOI:** 10.3389/fcell.2021.733066

**Published:** 2021-08-20

**Authors:** Luis B. Tovar-y-Romo, Alicia Guemez-Gamboa, João M. N. Duarte

**Affiliations:** ^1^Institute of Cellular Physiology, Universidad Nacional Autónoma de México, Mexico City, Mexico; ^2^Department of Neuroscience, Feinberg School of Medicine, Northwestern University, Chicago, IL, United States; ^3^Wallenberg Centre for Molecular Medicine, Faculty of Medicine, Lund University, Lund, Sweden

**Keywords:** neuronal death mechanisms, neuronal recovery, neuroinflammation, transcriptome, axon regeneration, CNS injury and repair, autophagy, neurorestoration

The central nervous system (CNS) is highly plastic, allowing for the integration of new neuronal circuits and vascular networks. This capacity to dynamically change brain structures upon external stimuli lays the groundwork for the adaptive transformations that help shape the many brain functions. However, the mammalian brain and spinal cord have minimal capacity for self-renewal, notably lacking robust mechanisms of adaptation to injury and reduced flexibility in terms of tissue repair and healing.

In this Research Topic published across Frontiers in Cell and Developmental Biology and Frontiers in Cellular Neuroscience, a collection of articles describing the most recent findings into the mechanisms that drive the physiological adaptation to neuronal injury draw a general picture of the current understanding of cellular and molecular processes involved in neuronal injury, neuronal regeneration, and restoration of brain functions.

Animal species better suited for investigating neural plasticity help us understand some of the primary mechanisms for neuronal regrowth. In this collection, two articles describe molecular processes for regeneration in zebrafish. First, Demirci et al. made a comprehensive description of transcriptional changes in the recovery phase of telencephalon damage; this comparative transcriptome analysis of the reorganizing telencephalon provides biological targets for assessing traumatic brain injuries in humans. Then, Shimizu et al. explored radial glia proliferation in neurogenic niches by performing histological analyses that revealed increased isolectin B4-positive macrophages before radial glia proliferation and identified IL6/Stat3 signaling as a trigger of radial glia activation during tectum regeneration.

Understanding the transcriptional profile of these processes is also relevant in mammalian systems. Here, Chohan puts into perspective how interventions based on the endogenously coded machinery for adult neurogenesis could provide a means to develop therapies. These therapies include the transplantation of gene-edited neuronal precursors for achieving neural repair in CNS injury and disease. Approaches like these enhance our understanding of the overall impact of directed therapies to recover function, as shown by Jermakowicz et al., who tested whether low-frequency electrical stimulation of the rat nucleus raphe magnus after incomplete spinal cord injury reversed damage. In this setting, the non-specific serotonin 5-HT_7_ receptor antagonist pimozide alleviated the initial transcriptional response of the injured spinal cord by stimulating the raphe magnus.

Besides transcriptional regulation, the control of the metabolic process is also a relevant mechanism to protect the brain from different types of insults where autophagy might play a key role. Torres-Esquivel et al. proved that alternative energetic substrates to glucose, such as the ketone body β-hydroxybutyrate, mechanistically help the brain engage the autophagic flux to prevent neuronal death by modulating the AMPK pathway. In another study using cultured spinal cord motor neurons as a model, Chen et al. described that autophagy inhibition increases the injury caused by oxygen and glucose deprivation. Both studies indicate that the proper activation of autophagy is needed to prevent the neuronal death elicited by metabolic stress.

The regulation of metabolic signaling can also improve neurological functions after damage. Bader et al. tested the treatment potential of glucagon-like peptide 1 (GLP-1) and glucose-dependent insulinotropic polypeptide (GIP) mimetics in a murine model of traumatic brain injury. They found that both a GLP-1 analog and a dual agonist of GLP-1 and GIP receptors improved cognitive deficiencies, neurodegeneration, and neuroinflammation, which were mechanistically mediated through the regulation of the PKA pathway.

The classical mechanisms of regulation of neural damage and recovery have also been explored in detail in this Research Topic, with new insights into the role of mitochondrial regulation of neuronal death. Benítez-Rangel et al. characterized the molecular responses of caspase-3 activation under different death inducers in a model of cerebellar granule neurons. They showed that voltage-gated Ca^2+^ channels in the endoplasmic reticulum are critical to maintaining mitochondrial membrane polarization.

As well, axon regeneration, regulation of vascularization, and neuroinflammation were given extensive consideration. Chung et al. reviewed the literature on the role of growth-associated protein-43 and brain acid-soluble protein 1 in neurodegenerative diseases. Both proteins present with alterations in their expression and phosphorylation profiles, highlighting both their involvement in neural injury response and regeneration, and their therapeutic potential to compensate for neuron loss. Jara et al. reviewed how CNS circuit connectivity after damage is enhanced by electrical stimulation by activating genetic neuroplasticity programs, revealing its therapeutic potential to enhance neuronal repair and recovery. Sutherland and Geoffroy summarize the evidence of axon growth decline with aging and evaluate how the cumulative alterations in astrocytes and microglia contribute to the impaired axonal repair with age. Sobrido-Cameán et al. examined the effect of inhibiting gamma-secretase on axonal regeneration after spinal cord injury in the sea lamprey. The authors used two different transcriptomic assessments with two related-drug applications targeting GABA receptors, suggesting gamma-secretase targeting as an effective axonal repair approach.

Regulation of brain perfusion is crucial for matching neuronal energetic demands. Regarding cerebrovascular regulation, Filchenko et al. explored the critical role of caveolin in the evolution of edema in stroke and its relation to the expression of aquaporin-4. The authors showed that loss of caveolin-1 results in decreased aquaporin-4 expression and contributes to brain swelling. Hierro-Bujalance et al. demonstrated that erythropoietin administration in a model of germinal matrix-intraventricular hemorrhage reduced long-term brain atrophy and malfunction. Vanherle et al. made a case for linking cardiovascular disease to cognitive impairment and dementia, where the diminished cerebral blood flow potentially induces neuronal death, especially in hypertension, heart failure, and stroke.

Finally, on the regulation of neuroinflammation, Jung and colleagues critically discussed the literature on the role of TNF-α-mediated neuroinflammatory processes and the potential of immunomodulatory imide drugs targeting the 3'UTR of TNF-α to reduce inflammation-associated neurodegenerative processes. The authors situate these compounds as potential inductors to alleviate symptoms and slow down the progression of neurodegenerative disease. Hu et al. investigated the regulatory effects of miR34a on the expression of major histocompatibility complex class I in hippocampal neurons and its involvement in shaping the neural morphology during development.

Despite not providing an exhaustive overview of the mechanisms that can be used for neuronal repair upon injury, this Research Topic collects original research articles and literature reviews that are representative of highly active research domains within the overall purpose of regenerating and/or repairing neurons for reestablishing adequate neuronal function.

## Author Contributions

All authors contributed to editing the Research Topic and prepared the Editorial piece.

## Conflict of Interest

The authors declare that the research was conducted in the absence of any commercial or financial relationships that could be construed as a potential conflict of interest.

## Publisher's Note

All claims expressed in this article are solely those of the authors and do not necessarily represent those of their affiliated organizations, or those of the publisher, the editors and the reviewers. Any product that may be evaluated in this article, or claim that may be made by its manufacturer, is not guaranteed or endorsed by the publisher.

